# Abundance, distribution, mobility and oligomeric state of M_2_ muscarinic acetylcholine receptors in live cardiac muscle

**DOI:** 10.1016/j.yjmcc.2013.01.009

**Published:** 2013-04

**Authors:** Tatiana A. Nenasheva, Marianne Neary, Gregory I. Mashanov, Nigel J.M. Birdsall, Ross A. Breckenridge, Justin E. Molloy

**Affiliations:** a Division of Physical Biochemistry, MRC National Institute for Medical Research, Mill Hill, London NW7 1AA, UK; b Division of Developmental Biology, MRC National Institute for Medical Research, Mill Hill, London NW7 1AA, UK

**Keywords:** Single molecule, TIRF, Acetylcholine receptor, Muscarinic, Cardiomyocyte

## Abstract

M_2_ muscarinic acetylcholine receptors modulate cardiac rhythm via regulation of the inward potassium current. To increase our understanding of M_2_ receptor physiology we used Total Internal Reflection Fluorescence Microscopy to visualize individual receptors at the plasma membrane of transformed CHO^M2^ cells, a cardiac cell line (HL-1), primary cardiomyocytes and tissue slices from pre- and post-natal mice. Receptor expression levels between individual cells in dissociated cardiomyocytes and heart slices were highly variable and only 10% of murine cardiomyocytes expressed muscarinic receptors. M_2_ receptors were evenly distributed across individual cells and their density in freshly isolated embryonic cardiomyocytes was ~ 1 μm^− 2^, increasing at birth (to ~ 3 μm^− 2^) and decreasing back to ~ 1 μm^− 2^ after birth. M_2_ receptors were primarily monomeric but formed reversible dimers. They diffused freely at the plasma membrane, moving approximately 4-times faster in heart slices than in cultured cardiomyocytes. Knowledge of receptor density and mobility has allowed receptor collision rate to be modeled by Monte Carlo simulations. Our estimated encounter rate of 5–10 collisions per second, may explain the latency between acetylcholine application and GIRK channel opening.

## Graphical abstract

Individual M_2_ muscarinic receptors were visualized and tracked in live cardiac tissue slices to establish their abundance, distribution, mobility and oligomeric state in situ.

**Figure:**
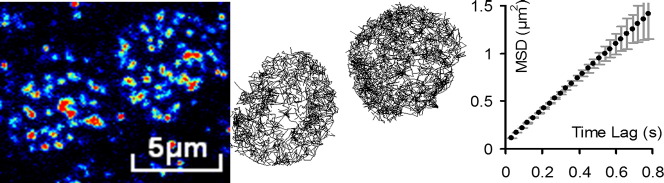

**Highlights:**

► The first observation of single molecules moving on the surface of living tissue ► Only ca. 10% of murine cardiomyocytes expressed M_2_ receptors at appreciable levels. ► M_2_ density at birth was 3-fold higher than that in embryonic and adult myocytes. ► M_2_ receptors were primarily monomeric but formed reversible dimers. ► M_2_ receptors diffused 4-times faster in heart slices than in cultured myocytes.

## 1 Introduction

Basal heart rate is regulated by cardiac pacemaker cells under the influence of cholinergic, parasympathetic, vagal stimulation. Acetylcholine (ACh) acts via the M_2_ subtype of muscarinic receptors, which are canonical G-protein coupled receptors (GPCRs) with 7-transmembrane spanning alpha-helices [1]. They bind a heterotrimeric (αβγ) G-protein on their intracellular face and the ligand, ACh, at the extracellular side. ACh binding causes the βγ G-protein subunits to activate inwardly rectifying, hetero-tetrameric [2] potassium GIRK channels [(Kir3.1)_2_ + (Kir3.4)_2_], increasing their potassium current and hyperpolarizing the cell membrane. This prolongs the electrical oscillation period of the pacemaker cell which slows the heartbeat. Patch clamp studies have shown that coupling between ACh binding to M_2_ receptors and downstream GIRK channel opening works via an “in-membrane” mechanism i.e. via direct interaction between the membrane bound G-protein βγ subunits and the integral-membrane GIRK channel [3–6]. The activation pathway therefore requires diffusion and collision of at least two membrane bound protein partners. Since the diffusional encounter rate contributes to the delay in GIRK channel conductance change following ACh stimulation [7,8] it is important to know the distribution, density, mobility and oligomeric state of the M_2_ receptors at the plasma membrane.

Previously, the surface density and tissue distribution of M_2_ receptors, the predominant ACh receptor subtype expressed in cardiac muscle [9], has been estimated using bulk methods such as quantitative antibody labeling [10–12]. A limitation of these methods is that they require high specificity of labeling [13] and assume homogenous receptor expression in all cells. The approach taken here has been to use a single molecule, fluorescence-based, method to directly observe and count M_2_ receptors in individual cells and live tissue samples using light microscopy. Inter alia, this approach also enables receptor density, mobility, and oligomeric state to be measured by direct observation.

Total internal reflection fluorescence (TIRF) microscopy uses an obliquely-angled incident laser beam to produce an evanescent field that penetrates just 100 nm beyond the microscope coverslip surface into the biological specimen [14]. This means that if a receptor protein is labeled with a suitable fluorophore it appears as a discrete spot of light that can be pinpointed and tracked as it diffuses within the lipid membrane. Recently, we applied TIRFM to visualize individual M_1_ receptors on the plasma membrane of mammalian cells, grown in culture [15]. By analyzing fluorescent spot intensities and spatial trajectories it was shown that M_1_ receptors underwent transient dimerization. The goal of the present study has been to apply similar methods to live tissue sections obtained from mouse cardiac muscle in order to visualize individual M_2_ receptors in their native context.

Various cell lines were used to check the binding specificity of the fluorescent ligands used to investigate the properties of the M_2_ receptors. A clonal CHO cell line, stably transfected with M_2_ receptors, allowed comparison with previous data obtained for CHO cells expressing M_1_ receptors [15]. An immortalized atrial cell line, HL-1 [16], which natively expresses M_2_ receptors [17], was also studied to see if the receptor properties were dependent on the cellular background. The results from these cell lines were compared with those from the investigations of primary dissociated cardiomyocytes and freshly dissected tissue slices from mouse heart.

We report, for the first time, that TIRF microscopy has enabled individual molecules to be counted and tracked on the plasma membrane of a tissue slice. The biophysical properties of individual M_2_ receptors in cardiac muscle slices have been quantitated at different stages of fetal and neonatal development. In addition, M_2_ receptors were shown to form reversible dimers on the surface of cells. The new ability to study single molecules in freshly dissected heart slices opens the future possibility to directly visualize other molecules involved in the ACh signaling cascade in cardiac cells.

## 2 Materials and methods

### 2.1 Radioligand binding assays

CHO cell culture, membrane preparation and the [^3^H]-NMS binding assays were as described previously [18] with minor modifications detailed in Supplementary information.

### 2.2 Fluorophore labeling procedure

The labeling conditions were designed to ensure rapid, maximal labeling of the M_2_ receptors with minimum background fluorescence [15]. This was facilitated by the very high affinity of Cy3B–telenzepine which enabled > 98% labeling of the receptors by the use of 1 to 10 nM ligand (50–500 times its K_d_) (see SD for full details).

### 2.3 TIRF imaging and image analysis

The TIRF microscope system and image analysis procedures have been described in detail previously [15,19,20] (see SD for full details).

### 2.4 Confocal microscopy

Confocal microscopy was performed at 23 °C using a Leica SP5-II confocal microscope (Leica Microsystems, UK) equipped with GaAsP hybrid detector, 488 nm and 561 nm lasers and 20 × NA 1.0 water-dipping objective lens giving a magnification of 200 nm per pixel in the x–y plane and using 1 μm z-steps to acquire volume sections.

## 3 Results

### 3.1 Characterization of fluorescent ligand binding to M_2_ receptors

CHO cell lines transfected singly with each of the muscarinic receptor subtypes (M_1_–M_5_) have been used for many years for the characterization of the binding and functional properties of individual muscarinic receptor subtypes and have been shown to accurately reflect the binding properties of those receptors in whole tissue [21]. Radioligand binding studies on membranes from CHO^M2^ cells showed that M_2_ muscarinic receptors bound Cy3B–telenzepine with a K_d_ of ~ 45 pM (log affinity 10.35 ± 0.02, n = 3, Fig. S1), comparable to the affinity for M_1_ receptors in CHO^M1^ cells [15]. Alexa488–telenzepine bound to M_2_ receptors with a 30-fold lower affinity than found for Cy3B–telenzepine (log affinity 8.83 ± 0.02, n = 2, Fig. S1), but still with nanomolar potency. This allowed > 85% of the receptors to be labeled using 10 nM fluorescent ligand. In comparison, 10 nM Cy3B–telenzepine labels > 99.5% of the M_2_ receptors. The inhibition curves (Fig. S1), show no evidence of cooperative or allosteric behavior and are compatible with a 1:1 stoichiometry of M_2_ receptor and ligand, as found for the same ligands with M_1_ receptors [15].

### 3.2 Evidence that M_2_ muscarinic receptors are specifically labeled by Cy3B–telenzepine

Wild type CHO cells do not natively express muscarinic receptors and we found no fluorescence signal when wild-type cells were incubated with Cy3B–telenzepine (using our standard labeling protocol). Furthermore, preincubation of the CHO^M2^ cell line with 10 μM of the potent muscarinic antagonist, atropine (which binds at all muscarinic receptor subtypes with ~ 1 nM K_d_), was sufficient to block Cy3B–telenzepine binding. Following atropine treatment, there was no cell-specific labeling although a small number of non-specifically adhered fluorophores were readily identified because they were completely immobile. These control experiments confirmed that the overwhelming majority of mobile fluorescent spots observed in our later experiments (described below) were muscarinic receptors and the small number of static spots was due to nonspecific fluorophore binding (probably to the coverslip surface). The only muscarinic receptor subtype present in the CHO^M2^ cells [22] and the predominant muscarinic receptor subtype present in the HL-1 cell line [17] and rodent cardiac cells [9,23,24] are M_2_ receptors, so we conclude that our data arises from specific labeling of the M_2_ receptor subtype (see SD and Fig. S3 for additional control experiments).

**Fig. 1:**
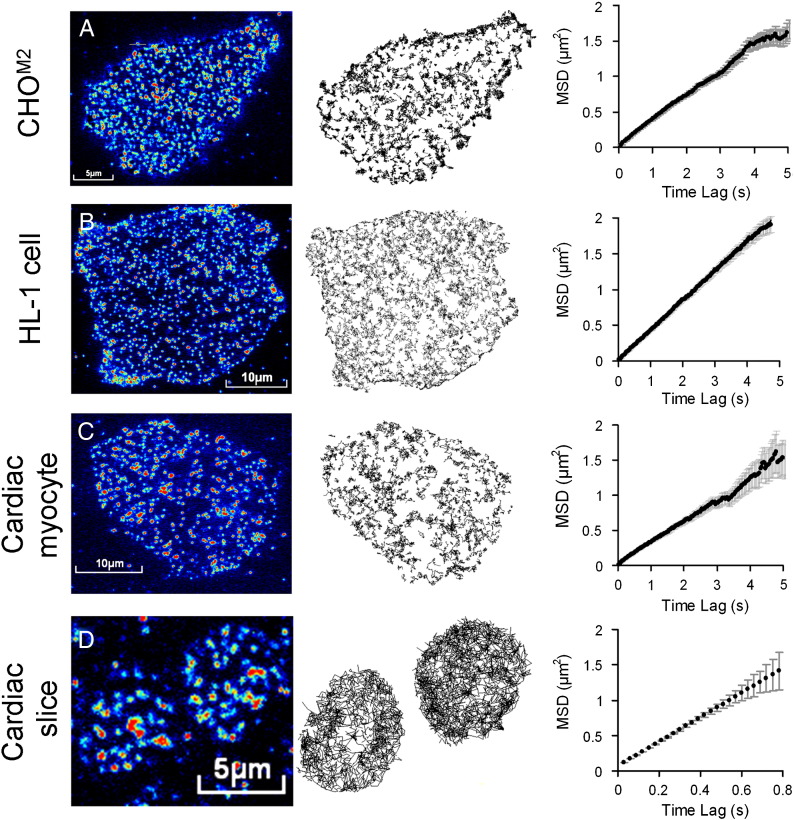
Mobility analysis of M_2_ receptors imaged under TIRF microscopy at 23 °C. M_2_ receptors in (A) CHO^M2^ cells, (B) HL-1 cells, (C) cardiac myocytes, and (D) cardiac slices were labeled with Cy3B–telenzepine and imaged as described in Materials and methods and SD. Left panels show images taken at the beginning of the recording.; middle panels show individual trajectories of fluorescent spots (809 in A, 1291 in B, 613 in C and 381 in D) identified in the cells; right panels show mean squared displacement (MSD ± SD) versus Δt (time interval) plots for the averaged M_2_ trajectories. Note the different scale on the abscissa in D, relative to A, B and C.

When viewed by TIRF microscopy, CHO^M2^ cells labeled with Cy3B–telenzepine appeared similar to CHO^M1^ cells. M_2_ receptors could be identified as individual fluorescent spots moving rapidly on the plasma membrane (Fig. 1A, Fig. S2 and Video SV1). The intensity profile of single spots had the expected, diffraction limited, spot size (~ 300 nm full width at half height) and had a sufficiently high signal-to-noise ratio to be reliably identified and tracked by an automated image analysis routine (See SD and Fig. S2A). The mean intensity of the majority of spots (Fig. S2B) was similar to that found for mono-dispersed, individual, Cy3B fluorophores viewed under identical conditions on a coverslip in vitro (Fig. S2B). Finally, the analysis of individual intensity trajectories over time demonstrated that the majority showed single step photobleaching (Fig. S2B). However, a significant population (40%) showed more complex behavior in which intensity alternated between two intensity levels.

### 3.3 M_2_ receptors visualized in CHO^M2^, HL-1 cells and isolated cardiomyocytes

The CHO^M2^ receptor density, determined from the number of fluorescent spots present on the cell membrane, was ~ 3 μm^− 2^ similar to bulk estimates calculated from the radioligand binding assays (see Fig. 1, SD, and Fig. S4). Analysis of individual receptor spatial trajectories (“tracks”) allowed the averaged mean squared displacement to be plotted as a function of time interval (Δt). The linearity of the MSD vs. Δt plots is consistent with receptors undergoing a Brownian random walk, characteristic of free diffusion within the lipid bilayer (Fig. 1A). The gradient of these plots gave an estimated D_lat_ ~ 0.1 μm^2^·s^− 1^, close to the value measured for M_1_ receptors in CHO^M1^ cells [15]. There was no evidence of receptor clustering or of cell regions deprived of moving fluorescent spots. Atrial derived HL-1 myocytes which natively express M_2_ receptors were labeled with Cy3B–telenzepine and examined by TIRFM. All cells were labeled (Fig. 1B, and Video SV2) to approximately the same extent and the distribution of individual fluorescent spot intensities was similar to that found on CHO^M2^ cells, indicating that they resulted primarily from a single fluorophore. The average receptor density was 7.5 μm^− 2^, and D_lat_ was 0.13 μm^2^·s^− 1^. There was no evidence for anomalous diffusion, or receptor clustering (See SD, Table 1, and Fig. S2).

**Fig. 2:**
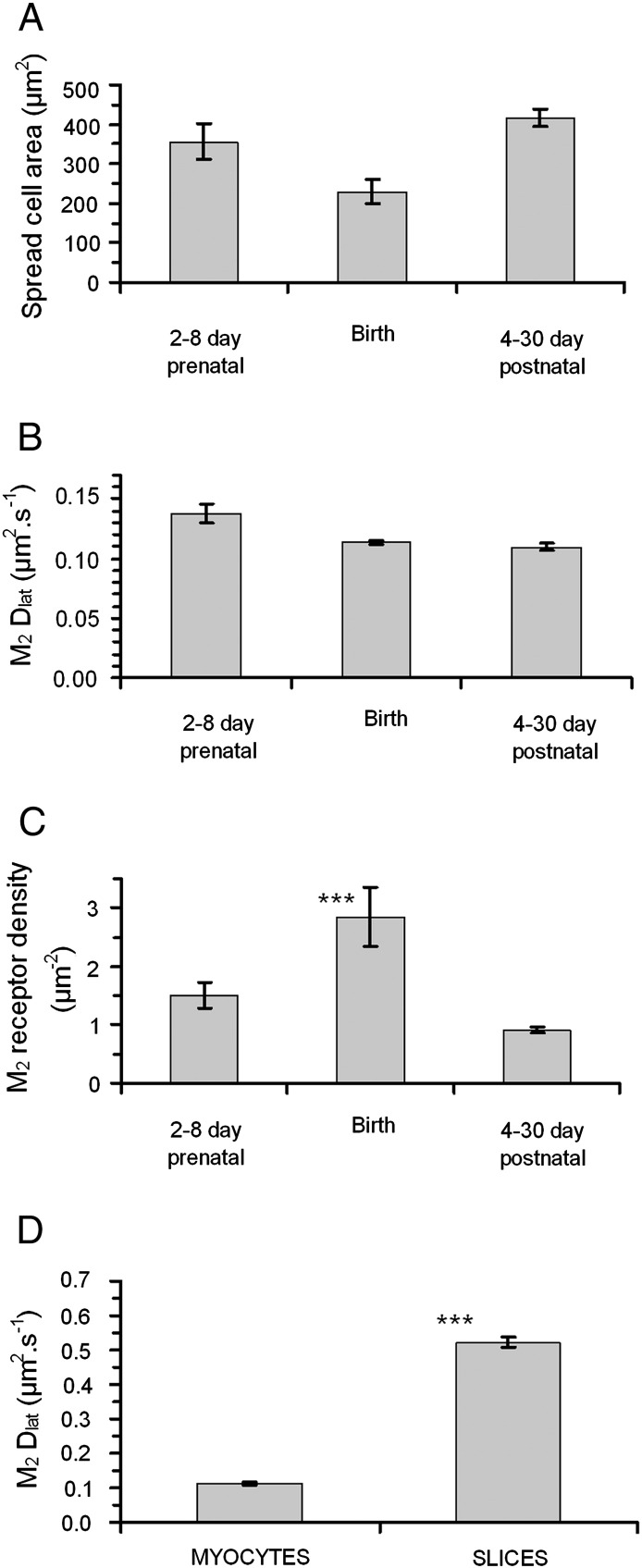
Properties of cardiomyocytes expressing M_2_ receptors during mouse development. (A) Average surface area of M_2_ expressing cardiomyocytes in primary cell culture (2–4 h after extraction). (B) There was no evidence for systematic changes in the mobility of M_2_ receptors in isolated cardiomyocytes during pre- and post-natal development. Furthermore, in all samples investigated the MSD vs Δt plots (see Fig. 1) were linear indicating that receptors diffused freely in the plasma membrane. (C) The density of M_2_ receptors on myocyte membranes at different ages. There is a significant increase in receptor density at birth; (***) indicates P < 0.01, using Students *T* test of “Birth” data compared with “pre-” and “post-” natal data. (D) M_2_ receptor mobility is significantly higher in freshly isolated heart slices than in primary cell cultures of cardiomyocytes; (***) indicates P < 0.01, using Students *T* test to compare the two samples.

Freshly dissociated cardiomyocytes, from mouse embryos and new-born mice were labeled as before and observed by TIRFM (example shown in Fig. 1C horizontal panels and Video SV3). We noticed that, unlike cultured CHO^M2^ and HL-1 cells, where 100% of the cells were labeled, only ~ 5–10% of the cells visible under bright-field illumination expressed M_2_ receptors. Neither the average cell spread area (visible under TIRF illumination) nor the M_2_ receptor mobility changed significantly with mouse age (from 8 days prenatal to 30 days postnatal) (Figs. 2A and B and Table 1). In contrast, receptor density increased significantly around the time of birth. At 2–8 days prenatal, receptor density was ~ 1 μm^− 2^ increasing to ~ 3 μm^− 2^ at birth and returning to ~ 1 μm^− 2^ at times greater than 4 days postnatal (Table 1 and Fig. 2C).

### 3.4 Temperature dependence of M_2_ receptor mobility

**Fig. 3:**
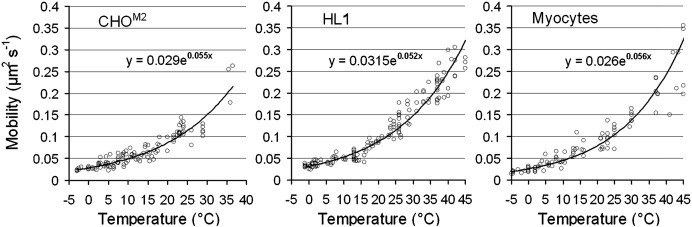
Graphs showing the change in average mobility (D_lat_) of M_2_ receptors as a function of temperature. For all cell types tested we found that mobility increased by a factor of 2 for each 10 °C increase in temperature. The lines are the best fit to a monoexponential function (see equations on the graphs).

We investigated the temperature dependence of receptor mobility in a series of experiments in which the microscope specimen chamber and objective lens were temperature controlled using a Peltier effect cooling/heating system. The temperature of the solution within the flow cell (i.e. the cell culture medium) was monitored using a thermocouple. Temperature was varied over the range − 4.5 °C to + 45 °C by changing the electrical current applied to the Peltier device and video records (of about 20 second duration) of cells labeled with the Cy3B–telenzepine were made at each temperature. Video records were then analyzed using the ASPT software (www.nimr.mrc.ac.uk/gmimpro/) and within each video sequence between 200 and 1000 fluorophores were tracked to obtain an estimate of D_lat_ from the gradient of MSD vs Δt plots. Each D_lat_ estimate was then plotted as a single data value at the known sample temperature (Fig. 3). For all cell types studied, D_lat_ increased two-fold for each 10° centigrade rise in temperature.

### 3.5 M_2_ receptors visualized in heart slices using TIRFM

**Fig. 4:**
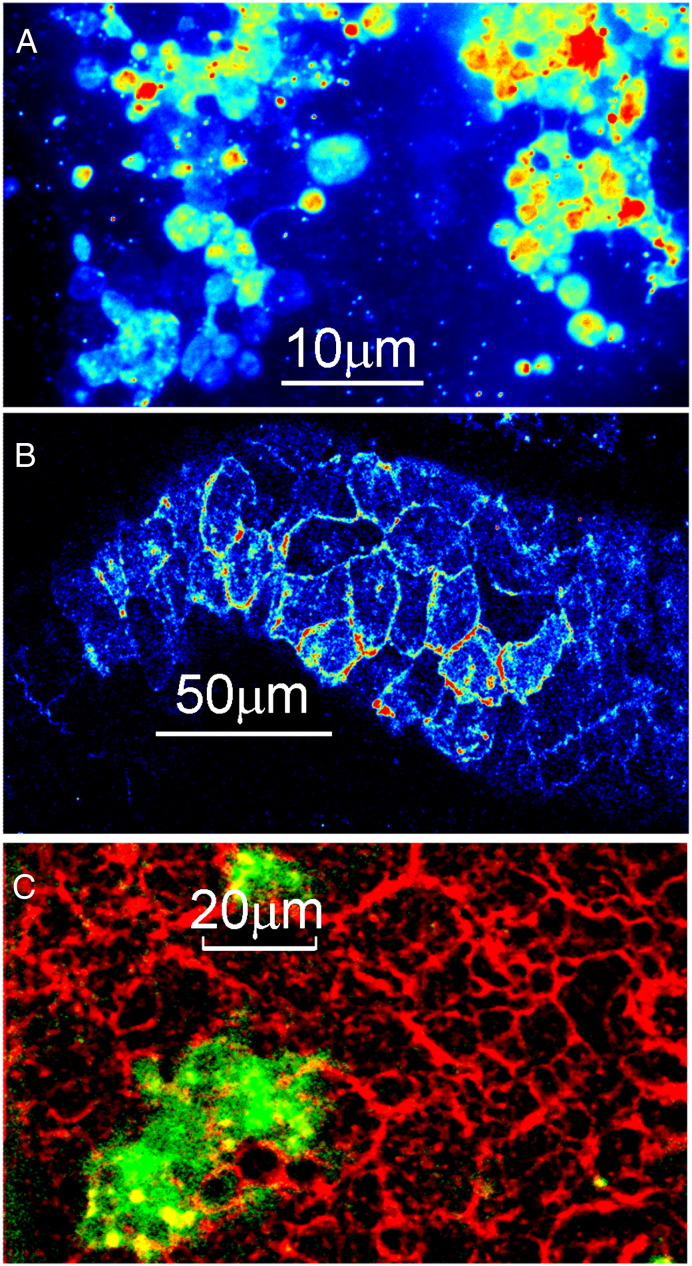
M_2_ receptors in live heart slices observed by TIRFM and confocal microscopy. (A) Region of a heart slice from a mouse embryo (5 days before birth) labeled with Cy3B-telenzepine and imaged by TIRF microscopy at 23 °C. The image, an average of 100 frames taken at the beginning of the recording (2 s total exposure time), is displayed using a pseudo-color intensity scale and shows clusters of M_2_ expressing cells surrounded by tissue containing many fewer M_2_ receptors. (B) Region of a slice from an adult mouse atrium labeled with Cy3B-telenzepine and imaged by confocal microscopy at 23 °C. It is displayed using a pseudo-color intensity scale and shows a cluster of M_2_ expressing cells. (C) Confocal image of a ventricle slice from a mouse embryo (2 days before birth). M_2_ receptors are labeled with Alexa488-telenzepine (green) and cell membranes are labeled with “DeepRed CellMask” (red).

Fresh cardiac tissue slices dissected from embryonic and new-born mouse heart were labeled and M_2_ receptors observed (as above) (see Fig. 1D left panel and Videos SV4 and SV5). A cluster of M_2_ expressing cells is also shown in Fig. 4A. Because the evanescent field produced by TIRF illumination only impinges on cell regions that are in close proximity to the microscope coverslip we observed circular cell “foot-prints” in which individual receptors could be readily identified and tracked (Fig. 1D center panel). The rate of receptor diffusion, D_lat_ ~ 0.6 μm^2^·s^− 1^, was significantly higher than for primary, cardiomyocytes of the same age mice (5 days prenatal) where D_lat_ ~ 0.17 μm^2^·s^− 1^ (Fig. 1D right panel and Fig. 2D).

### 3.6 M_2_ receptors visualized in heart slices using confocal microscopy

Since TIRF imaging only reveals cell regions that come in close proximity to the cover-slip (Figs. 1D, 4A), confocal microscopy was used to examine receptor distribution deeper into the cardiac tissue slices (Fig. 4B). There was distinct heterogeneity in the level of M_2_ labeling between cells seen within a single confocal Z-section. To explore this further, two-color imaging was used to confirm that M_2_ receptors labeled with Alexa488–telenzepine co-localized with membranes labeled with “DeepRed CellMask”. This showed that M_2_ receptors co-localized with membrane staining (Fig. 4C) but that some cell membranes were devoid of receptors or expressed receptors at very low levels.

### 3.7 Analysis of receptor stoichiometry in primary cardiomyocytes

In order to test whether M_2_ receptors exist as monomers, dimers or higher oligomers in primary cardiomyocytes, intensity trajectories were examined in more detail to see whether individual fluorescent spots showed multiple photobleaching steps. The integrated intensity of each spot was determined over a 5 × 5 pixel region (corresponding to 500 × 500 nm^2^) and expressed as the average counts per pixel (i.e. the total counts per object were 25 times larger). The intensity trajectory for each object, tracked over time as it moved at the plasma membrane, was saved along with the spatial (x, y) coordinates (determined with 25 nm precision by centroiding the spot images as above). Intensity trajectories were smoothed using a 3-point, running median filter and sudden changes in intensity were identified from peaks in the 1st derivative (calculated over a 5-point running window). Intensity trajectories were then fitted to a sequence of stepwise changes by calculating the mean of intensity values between each transition point. This allowed different trajectory “types” to be scored (Figs. S5, S6 and SD text). For example: “Type 1” trajectories remain at fairly constant intensity and then drop immediately to background within a single video frame (consistent with a single stepwise drop in intensity); “Type 2” trajectories showed two stepwise drops in intensity; “Type 3” exhibited an initial, constant level that increased to a higher level and then fell to zero (see SD and Fig. S6). We examined whether the incidence of "Type 1" trajectories decreased with receptor expression level, as would be expected if dimer formation were reversible. Our analysis showed that in perinatal cardiomyocytes (two days before birth, mean density of M_2_ receptors ~ 1.5 μm^− 2^) ~ 74% of the trajectories matched the type 1 pattern and ~ 7% matched the type 2 pattern. In myocytes from newborn mice (mean M_2_ density ~ 3 μm^− 2^) ~ 57% trajectories showed a type 1 pattern and ~ 10% matched the type 2 pattern.

## 4 Discussion

We report here, for the first time, the imaging of individual M_2_ muscarinic acetylcholine receptors in primary cardiac myocytes and cardiac tissue slices obtained from pre- and post-natal mice using TIRF microscopy. Previous studies have shown that the overwhelming majority of muscarinic receptors expressed in cardiac muscle are of the M_2_ receptor subtype [9,23,25]. In the present study, use of control cell lines (CHO, CHO^M2^ and HL-1) established that M_2_ receptors were specifically labeled with the fluorescent antagonist and individual fluorescent spots could be identified and tracked in a sequence of video images recorded at up to 50 frames per second using TIRF microscopy.

### 4.1 General findings

In all samples examined, we were able to identify fluorescently labeled receptors as discrete spots of light, most of which had an average intensity similar to monodisperse Cy3B dye molecules viewed under similar conditions. Fluorescently-tagged receptors were automatically identified, counted and tracked as they diffused within the plasma membrane using an image analysis system [20]. Receptor density was estimated by spot counting at densities below 0.8 μm^− 2^ and extrapolation based on the bulk photobleaching rate to obtain reliable estimates of density up to 10 μm^− 2^.

Detailed analysis of individual intensity trajectories showed that the majority of fluorescent spots underwent single-step photobleaching, which is the expected behavior of a single fluorophore. Furthermore, histograms of the intensity distribution of individual spots were consistent with the majority of spots arising from a single fluorophore (with a peak value around that found for monodispersed Cy3B molecules). The histogram had a tail which could be explained either by the chance overlap between two fluorophores (that were closer than the diffraction limit of the microscope, where inter-spot spacing < 350 nm or could be due to a minor population of dimeric M_2_ receptors ~ 20% of all spots at any instant in time).

The reported receptor densities observed in radioligand binding studies on the membranes of the CHO^M2^ cell line used in these studies allow a prediction of the M_2_ receptor density on the cell surface observed in the TIRF experiments. This value (3 μm^− 2^) is in agreement with the observed value shown in Table 1 (for calculations see SD).

The downstream analysis of spatial trajectories involved constructing MSD vs. Δt plots which enabled the type of diffusive behavior to be characterized and allowed the lateral diffusion coefficient, D_lat_, to be determined (from the gradient of the plot). The critical observation was that the MSD vs. Δt plots were linear over the timescale of our measurements and did not show either an upward inflection (characteristic of “diffusion with flow”) or a downward inflection (characteristic of “caged diffusion”) [26]. This means that M_2_ receptors undergo an unrestricted 2-D random walk, characteristic of free diffusion within the lipid bilayer. There was no evidence for clustering or uneven distribution of M_2_ molecules, in any of the specimens examined. This result contrasts with a fluorescence correlation spectroscopy (FCS) study that indicated β-adrenoreceptors cluster on the plasma membrane of cardiomyocytes [27].

### 4.2 Receptor diffusion is faster in cardiac tissue slices than in adherent, cultured cells

Whereas the rate of M_2_ receptor diffusion was similar in CHO^M2^, HL-1 and isolated primary cardiomyocytes (D_lat_ was in the range 0.11 to 0.17 μm^2^·s^− 1^ at 23 °C) it was significantly higher in cardiac tissue slices (D_lat_ ~ 0.6 μm^2^·s^− 1^ at 23 °C). We found no systematic change in D_lat_ within either primary myocytes or cardiac tissue slices obtained from animals aged 8 days prenatal to 30 days postnatal. Due to technical limitations, our studies on tissue slices were performed at 23 °C. We measured the temperature dependence of M_2_ mobility over the range − 5 °C to 45 °C for CHO^M2^, HL-1 and myocytes and found that for all cell types mobility approximately doubles when temperature is increased by 10 °C. Therefore, we predict that M_2_ mobility in heart muscle cells of live animals (~ 37 °C) will be around 1 μm^2^·s^− 1^. Since receptor movement is consistent with a simple Brownian walk we believe that the faster diffusion rate found in cardiac tissue slices compared to primary cardiomyocyte cultures could be due to reduced lipid mobility in the adherent culture cardiomyocytes compared to the fresh tissue slices. The finding is important because most cell biological and biophysical studies use adherent cultured cell lines.

### 4.3 Receptors are mainly monomeric but a sub-population of receptors are dimeric

Analysis of individual spot intensity trajectories shows that the majority (~ 60%) of telenzepine-labeled species present in primary myocytes exhibit single-step photobleaching and the simplest interpretation is that these receptors are monomeric. However, the remaining spots showed more complex intensity trajectories. The overwhelming majority of these trajectories exhibited alternation between two discrete intensity levels (see SD and Fig. S6B). This is consistent with either two unitary intensity spots coming close together (within 300 nm of each other) so their diffraction-limited images coalesced or there being a sub-population of dimeric receptors. Importantly, we found no evidence for higher oligomeric states (e.g. tracks or spots showing intensity levels corresponding to more than two fluorophores or stepwise intensity changes corresponding to 3 or more intensity levels). We conclude that the majority of M_2_ receptors in primary cardiomyocytes are monomeric with a smaller subpopulation existing as dimers for some of the time. Goin and Nathanson [28] have also reported, using the BRET technique, that M_2_ receptors are a mixture of monomers and dimers. The fact that intensity increases in tracks are seen (Fig. S6B) means that dimer formation is reversible on the ~ 1 second time scale as was found for M_1_ muscarinic receptors [15]. The duration of the tracks at “level 2” (i.e. double the expected fluorescence of a single fluorophore (see Fig. S6B)) is ~ 1 s. Monte-Carlo simulations show that the number of such observations (~ 10% of all tracks) greatly exceeds values expected due to chance overlap in the position of two fluorescently-tagged molecules that diffuse independently at 0.15 μm^2^·s^− 1^. Notably, the proportion of dimer track types (e.g. Type “2”,“3”,“6”) increased with increasing receptor density (see Results section 3.7) as expected for a first-order binding process governing dimer formation. However, detailed kinetic modeling of this observation is complicated by the fact there is also an increased “artefactual” coincidence of receptor localization due to chance overlap between the optical point spread functions at high fluorophore density. Notwithstanding that caveat, our observation is consistent with a dynamic equilibrium between monomeric and dimeric receptor states.

### 4.4 Expression of M_2_ receptors in cardiac tissue slices and primary cardiomyocytes is heterogeneous

We found that only a sub-population of primary cardiomyocytes (5–10% of all cells) expressed M_2_ receptors at the plasma membrane. Some cells, visible under the bright-field illumination used for cell counting, could be fibroblasts or other cell types present in mouse heart, thus the proportion of M_2_ expressing cells could be somewhat over 10% but this does not affect our main conclusion. This was also found in atrial and ventricular tissue slices where M_2_ muscarinic receptors were expressed in clusters of cells. Confocal imaging of tissue slices confirmed initial observations made using TIRF microscopy showing that many cells showed clear membrane staining yet were devoid of telenzepine labeled M_2_ receptors. This finding is inconsistent with immunohistochemical studies of muscarinic receptor distributions in cardiac muscle. However, studies using receptor gene-deficient mice have recently called into question the specificity of most of the antibodies and labeling approaches used [13]. More direct measures of receptor concentration in mouse heart gives values ~ 0.1 nmol·g^− 1^ membrane protein [23,29], equivalent to an average receptor density of ~ 0.2 μm^− 2^ (assuming all cells express M_2_ receptors at the same level). Our findings are consistent with this, given that we find ~ 10% of cells express receptors at a density of 2 μm^− 2^ (for calculations see SD).

### 4.5 Receptor density is highest at the time of birth

The density of M_2_ receptors increased dramatically and significantly (P < 0.1%) at the time of birth (~ 3 μm^− 2^) and decreased back to ~ 1 μm^− 2^, three days after birth. In contrast, the levels of the Kir3.1 and Kir3.4 subunits in mouse heart do not decrease after birth [30]. This suggests an important role for muscarinic receptor expression level in regulating the observed increase in acetylcholine-induced K^+^ currents during gestation and the decrease in basal heart rate around the time of birth [30].

### 4.6 Receptor diffusion and latency of GIRK channel signaling

Knowing the density and lateral mobility of M_2_ receptors in native cardiac cells we modeled receptor dynamics at the plasma membrane: We know that the peak M_2_ receptor density is ~ 3 μm^− 2^ and the majority of receptors are monomeric, diffusing within the plasma membrane in an unconstrained, random, fashion with D_lat_ of ~ 1 μm^2^·s^− 1^ estimated at 37°. The estimate of GIRK channel density in rabbit sino-atrial node is ca 0.7 μm^− 2^ [31], which is of the same order as the receptor levels we observe in the mouse heart.

A Monte-Carlo computer simulation of the receptor movement shows (Fig. S7) that receptors collide (i.e. approach within a distance of 6 nm) 5 to 10 times per second. If we assume GIRK channel density is similar to the M_2_ density then we would expect a diffusion-limited latency between acetylcholine binding to the receptor and downstream GIRK channel activation to be in the region of 150 ms. Collision theory indicates that collision rate depends on the summed concentrations and summed diffusion coefficients of the reactants so the effect of changing the mobility or density of one interacting partner will have a linear effect on collision rate.

Although factors such as G-protein recruitment, activation and dissociation, may be additional rate-limiting factors in the downstream signaling pathway, our findings indicate that receptor collision rate may explain the ~ 50–150 ms latency [7,8,31–34] measured between the application of ACh to rabbit sino-atrial node and the electrophysiological response. Precoupling of the G-protein subunits to the GIRK channel [35,36] has the potential to simplify the scheme to a bimolecular collision coupling between the acetylcholine-occupied receptor and the G-protein-coupled GIRK channel.

We have shown that the ratio of dimers to monomers is a function of expression level. The observed rapid rate of dimer–monomer interconversion of M_2_ receptors may tend to optimize the time-scale of the functional output by decreasing any differential effect of dimers versus monomers in their interaction with GIRK channels.

## Abbreviations: (All non-standard abbreviations/acronyms)

ACh: Acetylcholine
ASPT: Automated single particle tracking
CHO^M1^: Chinese hamster ovary cells stably transfected with the M_1_ receptor
CHO^M2^: Chinese hamster ovary cells stably transfected with the M_2_ receptor
GIRK: G-protein coupled, inwardly rectifying, potassium channel (Kir3.x or K(ACh))
GPCR: G-protein coupled receptor
M_1_: M_1_-subtype of the muscarinic acetylcholine receptor family
M_2_: M_2_-subtype of the muscarinic acetylcholine receptor family
TIRF(M): Total internal reflection fluorescence (microscopy)
D_lat_: Lateral diffusion coefficient (μm^2^·s^− 1^)
MSD: Mean-squared displacement (μm^2^)
∆t: Incremental time interval between measurements (s)
ρ: Surface density of receptor molecules (μm^− 2^)

## Author contributions

TAN, GIM & MN did the cell imaging work, NJMB contributed to the ligand binding assays; GIM built the TIRFM imaging system, wrote the software for data capture and image analysis; TAN, GIM, JEM, NJMB did the data analysis; RAB, NJMB & JEM designed and conceived the experiments; JEM, NJMB, GIM and RAB wrote the paper.

## Disclosures

None declared.

The following are the supplementary data related to this article.

Video S1 TIRFM video showing M_2_ receptors labeled with Cy3B-telenzepine moving on the plasma membrane of a CHO^M2^ cell.

Video S2 TIRFM video showing M_2_ receptors labeled with Cy3B–telenzepine moving on the plasma membrane of an HL-1 cardiomyocyte cell.

Video S3 TIRFM video showing M_2_ receptors labeled with Cy3B–telenzepine moving on the plasma membrane of a cultured primary cardiomyocyte isolated from freshly dissected mouse heart.

Video S4 TIRFM video showing M_2_ receptors labeled with Cy3B–telenzepine moving on the plasma membrane of a mouse heart slice (zoomed area, 5 days before birth, 33 frames s^− 1^).

Video S5 TIRFM video showing M_2_ receptors labeled with Cy3B–telenzepine moving on the plasma membrane of a mouse heart slice (ventricle, 2 days before birth, 50 frames s^− 1^).

Supplementary material.

## Figures and Tables

**Table 1:** Summary of the densities and D_lat_ values (lateral diffusion coefficient) of M_2_ receptors on CHO^M2^, HL-1, primary cardiomyocytes and cardiac tissue slices.

	CHO^M2^	HL-1	Myocytes	Heart slice	aCHO^M1^
**Density** **(μm^− 2^)** *(N_cells_)*	**3.1 ± 0.8**^b^ *(n = 16)*	**7.5 ± 2.8** *(n = 22)*	**1.8 ± 0.4** *(n = 6)*	**0.88 ± 0.22**^c^ *(n = 14)*	**2.1 ± 0.02** *(n = 9)*
**D_lat_(μm^2^**·**s^− 1^)** *(N_objects_; N_cells_)*	**0.109 ± 0.015** *(13613; 13)*	**0.126 ± 0.013** *(27809; 10)*	**0.165 ± 0.013** *(2712; 6)*	**0.63 ± 0.01** (3723; 14)	**0.097 ± 0.006** *(21954; 9)*

a From Hern et al., 2010 (7).

b Values stated in bold are *mean ± SD* values in italics indicate the sample size.

c These values are measured from the observed spots from slices of mouse hearts (5 and 2 days before birth). It was not possible to correct these values for the effect of photobleaching because the cell footprint is very small and molecules move fast into and out of the evanescent field. It is likely that the original density has been underestimated considerably, possibly by a factor of 2–3.
