# Supplementary figures and images for: Abundance, distribution, mobility and oligomeric state of M2 muscarinic acetylcholine receptors in live cardiac muscle
Source: J Mol Cell Cardiol. 2013 Apr;57:129–36. doi: 10.1016/j.yjmcc.2013.01.009 (PMC3605596; doi:10.1016/j.yjmcc.2013.01.009)

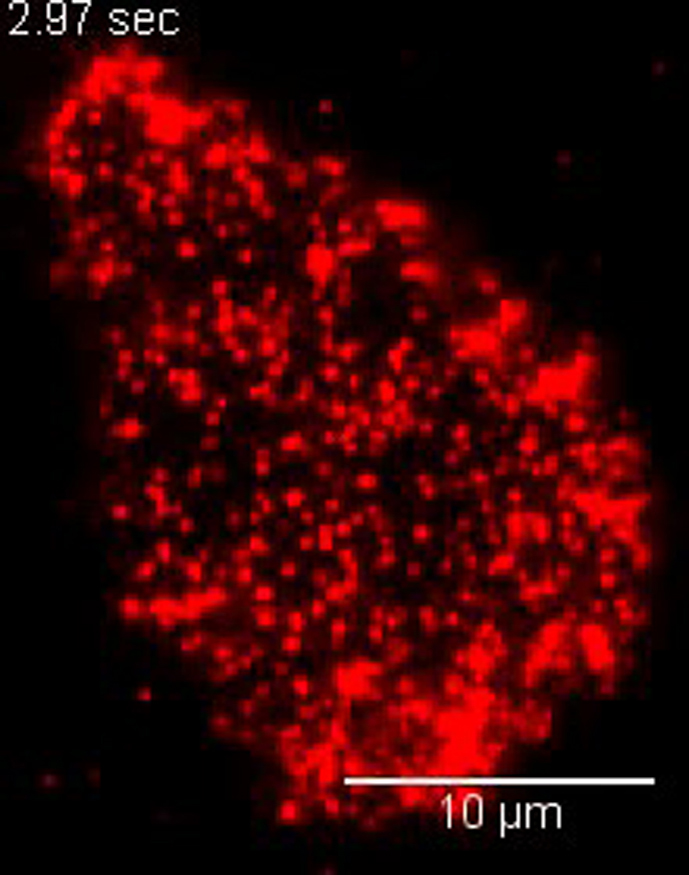

Supplement: Video S1 — TIRFM video showing M2 receptors labeled with Cy3B-telenzepine moving on the plasma membrane of a CHOM2 cell. [file mmc1.jpg]

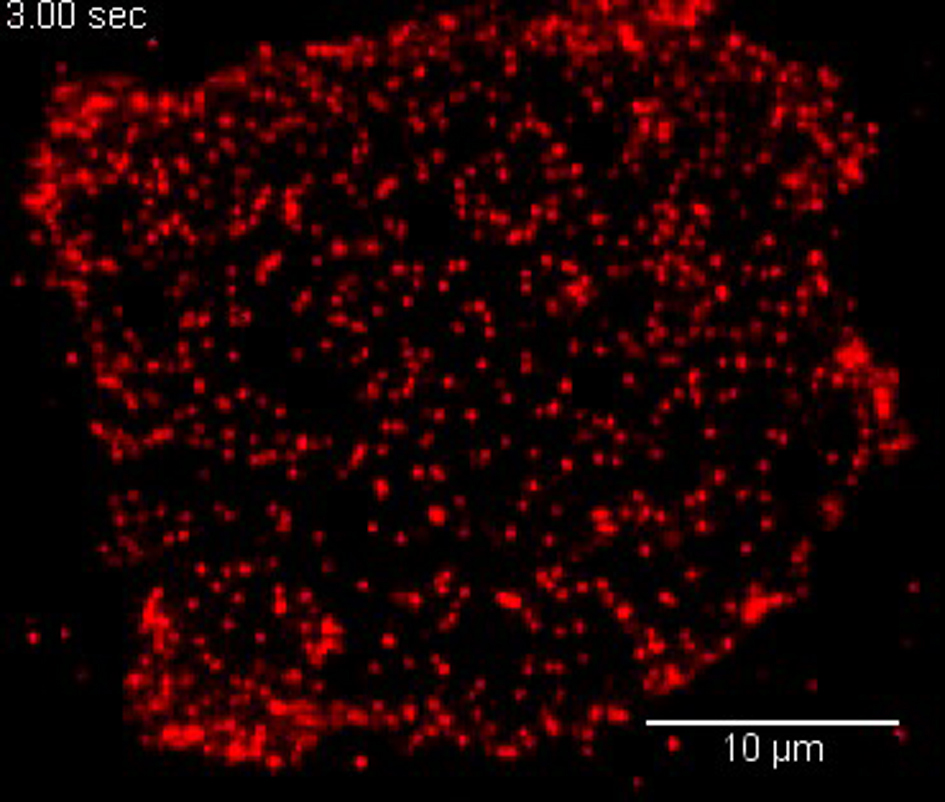

Supplement: Video S2 — TIRFM video showing M2 receptors labeled with Cy3B–telenzepine moving on the plasma membrane of an HL-1 cardiomyocyte cell. [file mmc2.jpg]

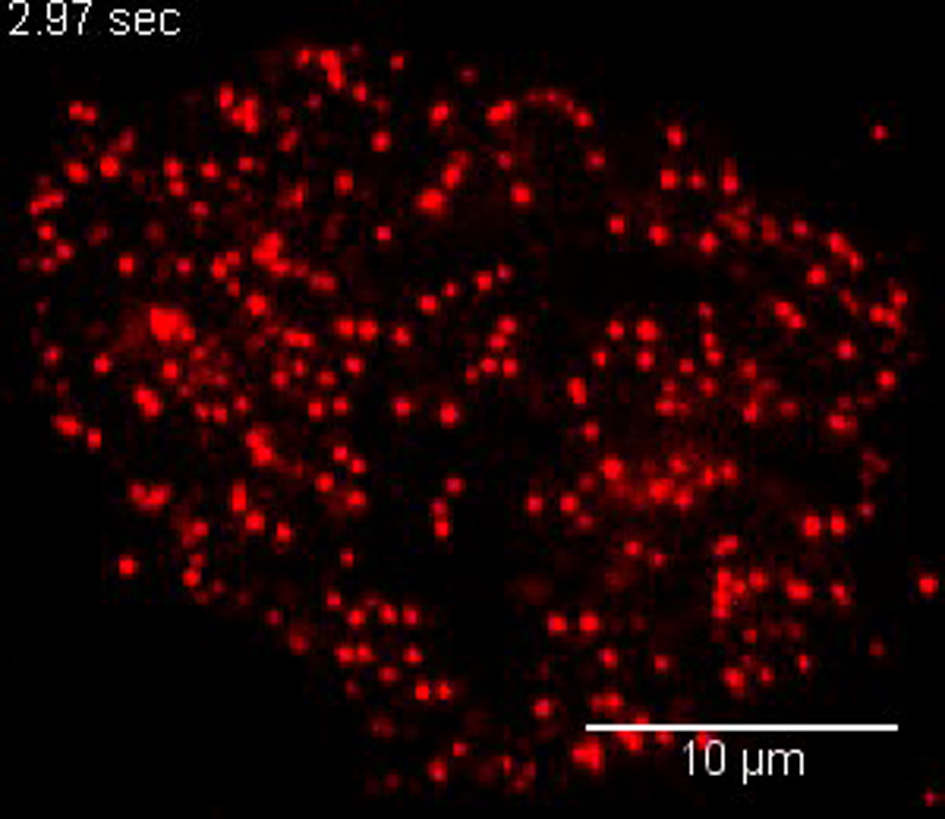

Supplement: Video S3 — TIRFM video showing M2 receptors labeled with Cy3B–telenzepine moving on the plasma membrane of a cultured primary cardiomyocyte isolated from freshly dissected mouse heart. [file mmc3.jpg]

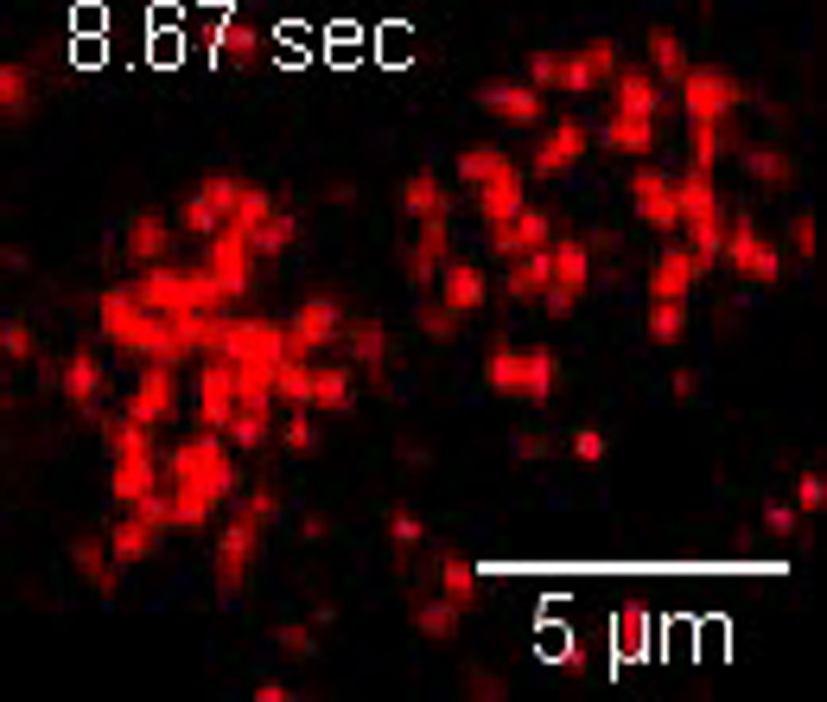

Supplement: Video S4 — TIRFM video showing M2 receptors labeled with Cy3B–telenzepine moving on the plasma membrane of a mouse heart slice (zoomed area, 5 days before birth, 33 frames s− 1). [file mmc4.jpg]

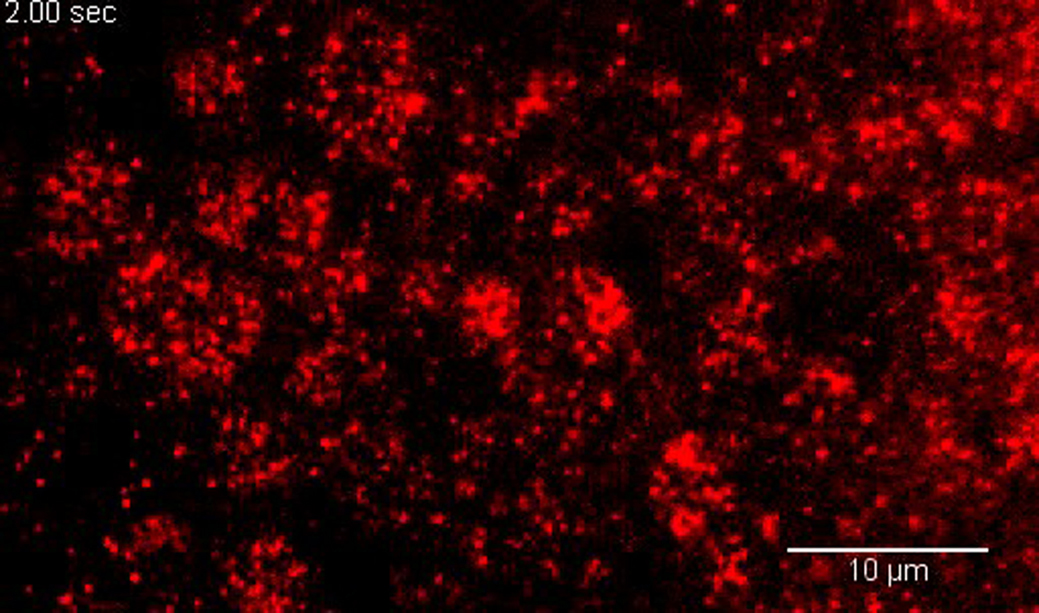

Supplement: Video S5 — TIRFM video showing M2 receptors labeled with Cy3B–telenzepine moving on the plasma membrane of a mouse heart slice (ventricle, 2 days before birth, 50 frames s− 1). [file mmc5.jpg]
