# Supplementary material for: Abundance, distribution, mobility and oligomeric state of M2 muscarinic acetylcholine receptors in live cardiac muscle
Source: J Mol Cell Cardiol. 2013 Apr;57:129–36. doi: 10.1016/j.yjmcc.2013.01.009 (PMC3605596; doi:10.1016/j.yjmcc.2013.01.009)
Supplement: Supplementary material. [file mmc6.doc]

**Abundance, Distribution, Mobility and Oligomeric State of M_2_ Muscarinic Acetylcholine Receptors in Developing Cardiac Muscle.**

Nenasheva *et al.*

**Supplementary Data**

**Supplementary Videos (see associated movie files)**

**SV1:** TIRFM video showing M_2_ receptors labeled with Cy3B-telenzepine moving on the plasma membrane of a CHO^M2^ cell.

**SV2:** TIRFM video showing M_2_ receptors labeled with Cy3B-telenzepine moving on the plasma membrane of an HL‑1 cardiomyocyte cell.

**SV3:** TIRFM video showing M_2_ receptors labeled with Cy3B-telenzepine moving on the plasma membrane of a cultured primary cardiomyocyte isolated from freshly dissected mouse heart.

**SV4:** TIRFM video showing M_2_ receptors labeled with Cy3B-telenzepine moving on the plasma membrane of a mouse heart slice (zoomed area).

**SV5:** TIRFM video showing M_2_ receptors labeled with Cy3B-telenzepine moving on the plasma membrane of a mouse heart slice.

**1. Experimental Procedures**

Chemicals and other materials were from Sigma-Aldrich, Poole, UK unless stated otherwise in the text.

***1.1. Cell culture and sample preparation***

A transformed cell line, CHO^M2^, which stably expresses M_2_ muscarinic receptors at a density of approximately 3 μm^­‑2^ was a kind gift of Prof. Noel Buckley (Kings College London) [[1](#_ENREF_1)] and an immortal atrial cardiac cell line, HL-1, were cultured as described previously [[2-4](#_ENREF_2)]. Hearts from C57/Bl10 embryos (up to -10 days), new-born mice and mouse pups (up to +30 days old) were dissected in ice-cold PBS and placed in an Eppendorf tube containing 500 µl of a collagenase solution (1 mg.ml^‑1^) at 37^o^C in a shaking incubator for 10 minutes. The collagenase was removed and the hearts washed in warm tissue culture medium 3 times. 500 μl of medium (Hanks solution +10% fetal bovine serum) was added and a p1000 Pasteur pipette used to disrupt the tissue and disaggregate the cells until there were no visible clumps of cells. Cells were plated onto cleaned 25mm diameter glass coverslips coated with fibronectin/gelatin for primary cell culture. Cardiac tissue slices were prepared from fresh murine heart using a scalpel blade. All mouse procedures were subject to institutional ethical review, and conformed to institutional welfare standards.

***1.2. Membrane preparation for radioligand binding assays***

CHO cell culture and membrane preparation were as described previously [[3](#_ENREF_3)]. Briefly, CHO cells stably expressing the cDNA encoding human muscarinic M_2_ receptors, termed CHO^M2^ cells, were grown in alpha-MEM medium (GIBCO) containing 10% (v/v) new born calf serum, 50 U/.ml^-1^ penicillin, 50 µg/.ml^-1^ streptomycin and 2 mM glutamine, at 37^o^C under 5% CO_2_. Cells were grown to confluence and harvested by scraping in a hypotonic medium (10 mM EDTA, 20 mM Hepes, *p*H 7.4). Sodium butyrate (5 mM) was added to the medium 24 hours before harvesting in order to increase receptor expression from ~1 pmol.mg^-1^ protein [[5](#_ENREF_5)] to ~3 pmol.mg^‑1^ protein [[3](#_ENREF_3)]. Membranes were prepared at 0^o^C by homogenization with a Polytron followed by centrifugation (40,000 x g, 15 min.), were washed once (0.1 mM EDTA, 20 mM Hepes, *p*H 7.4,) and were stored at -70^o^C in the same buffer at protein concentrations of 2-5 mg.ml^-1^. Protein concentrations were measured with the BioRad Protein Assay Reagent (which is based on the Bradford assay) using bovine serum albumin as the standard.

***1.3. [^3^H]-NMS binding assays***

[^3^H]-NMS was obtained from Amersham International, Chalfont St Giles, Buckinghamshire, UK. Frozen membranes were thawed, resuspended in incubation buffer (100 mM NaCl, 10 mM MgCl_2_, 20 mM Hepes, *p*H 7.4) and incubated in polystyrene deep-well plates with radioligand (0.44 nM) in the presence and absence of unlabeled fluorescent ligands (10^‑11^ to 10^‑6^ M) for six hours at room temperature in a volume of 1 ml. After filtering, washing twice with buffer (1ml/well), and drying at room temperature for ~40 hours at room temperature followed by 30 minutes at ~70^o^C, scintillant (Ultima Gold B, 40 μl/well) was added and the plates mixed for 5 minutes. Radioactivity was counted on a normalized, calibrated 1450 Microbeta Trilux counter (5 min. per sample). The membrane protein concentration (9 µg/well) was adjusted so that not more than about 10% of the added radioligand was bound. Nonspecific [^3^H]NMS binding was measured in the presence of 10 μM QNB (an antagonist with picomolar potency) and accounted for 1-5% of total binding. Total and nonspecific [^3^H]NMS binding to the membranes was also measured in the presence of a higher concentration of radioligand (2.6 nM). These data, together with the control data at the lower concentration of [^3^H]NMS, allowed ‘2-point’ calculations of the [^3^H]NMS affinity constant (K_d_ = 0.31 to 0.37 nM) and the receptor density (3.46 to 3.61 pmole.mg^-1^ membrane protein, n = 3) within each experiment. Data points were usually measured in duplicate. Fluorescent ligands were dissolved in dimethyl sulfoxide (DMSO) and further diluted in DMSO. The final DMSO concentration was 1% in all assays, a concentration which had no effect on the binding of the radioligand.

The IC_50_ values of the fluorescent ligands were estimated using Prism 4.0 and the equation for a simple inhibition curve, with the slope factor constrained to 1.0 (i.e. with the ligands binding to the receptor in a 1:1 stoichiometry with the radioligands). The IC_50_ values were converted to affinity values using the Cheng-Prusoff correction term derived from the determination of the [^3^H]NMS affinity constant and the [^3^H]NMS concentration used in the inhibition assays (see Fig. S1).


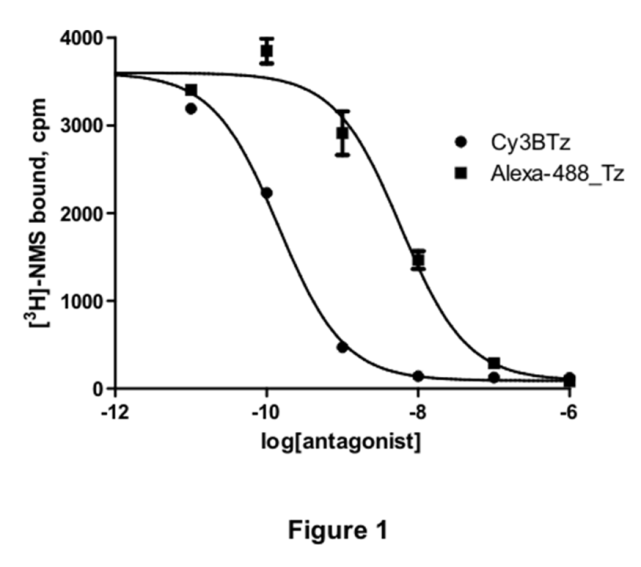


**Figure S1.** Inhibition of [3H]NMS binding to M_2_ muscarinic receptors on CHO^M2^ cell membranes by Cy3B-telenzepine (Cy3B-Tz) and Alexa488-telenzepine (Alexa488-Tz). The data shown are from a single experiment and the curves are the best fit inhibition curves for a 1:1 receptor interaction with the ligands.

***1.4. Fluorophore labeling procedure (cultured cells)***

CHO, CHO^M2^, HL-1 cells and primary cardiomyocytes were transferred to a balanced Hank’s salts solution (containing 10% fetal calf serum and 20 mM HEPES, *p*H 7.4) on fibronectin-coated N^o^ 1, 25 mm, round coverslips. (VWR, Leicestershire, UK). Cy3B-telezepine was added to the Hank’s medium to a final concentration of 1 or 10 nM. Cells were then incubated at 23°C for one hour. The cells were then gently washed, three times, with fresh Hank’s solution to remove unbound Cy3B telenzepine and the coverslips were assembled into a custom-made microscope viewing chamber [[6](#_ENREF_6)] that was refilled with Hank’s solution. The slow dissociation rate of Cy3B-telenzepine meant there was little loss of fluorescence labeling of the cells after washing away unbound fluorescent ligand. Pre-incubation with 10 µM atropine (a potent non-fluorescent muscarinic antagonist) for 2-3 hours, followed by incubation with Cy3B-telenzepine inhibited all labeling of the cells by the fluorescent antagonist. The tight binding and slow dissociation kinetics of the telenzepine ligand means that it is possible to observe fluorescently labeled receptors for many hours after sample preparation.

***1.5. Fluorophore labeling procedure (cardiac tissue slices)***

For TIRF and confocal imaging studies, freshly sectioned heart slices (0.5 to 1 mm thick) were washed in Hank’s buffer as described above and labeled for one hour in Hank’s solution containing Cy3B-telenzepine (10 nM). For dual-color confocal imaging, "CellMask, Deep Red” plasma membrane stain (Invitrogen, UK) was added to the labeling solution (final concentration 10 µg.ml^-1^) 10 minutes before the end of the one hour labeling procedure with Alexa488-telenzepine (10 nM) giving >85% labeling of the M_2_ receptors. Before viewing, tissue slices were gently washed, three times, in Hank’s solution and placed on a fibronectin coated No1 round coverslip within the imaging chamber. A fine nylon mesh (0.5×0.5 mm^2^ aperture size), stretched across a stainless steel tambour, was placed over the tissue slice in order to hold it against the coverslip surface. The imaging chamber was filled with Hank’s solution and placed on a microscope stage for either TIRFM or confocal imaging.

***1.6. TIRF Imaging system***

The beam from a 100 mW, 556 nm laser (MGL-556–100, Suwtech, China) was expanded by a Galilean beam expander and focused at the back focal plane of a high numerical aperture objective lens (AlphaPlan, 100x, NA 1.45, Carl Zeiss) [[6](#_ENREF_6), [7](#_ENREF_7)]. A front-surface silvered mirror (3 mm diameter) was used to direct the laser beam into the objective lens by positioning it immediately below, and at the extreme edge, of the back aperture. The average laser intensity at the specimen plane was ~40 μW.μm^‑2^. The incident laser beam angle was adjusted to 64° to create the evanescent field at the glass-water interface. A digital EMCCD camera (iXon897BV, Andor, UK) was used to acquire video sequences that were stored directly on a computer hard drive using a computer frame-grabber card and proprietary software. The microscope image magnification was calibrated using a reticule; giving 100 nm per pixel in both x and y camera axes. Experiments were performed at 23°C and video records were collected at either 33 or 50 frames.s^‑1^ (except where noted in the text).

***1.7. Appearance of cells labeled with Cy3B-telenzepine***

*(this text is repeated from the main manuscript for clarity)* When viewed by TIRF microscopy, CHO^M2^ cells labeled with Cy3B-telenzepine appeared similar to CHO^M1^ cells [[7](#_ENREF_7)]. M_2_ receptors could be identified as individual fluorescent spots moving rapidly on the plasma membrane (Fig. S2 and Movie SM1). The intensity profile of single spots had the expected, diffraction limited, spot size (~300 nm full width at half height) and had a sufficiently high signal-to-noise ratio to be reliably identified and tracked by an automated image analysis routine (Fig. S2A). The mean intensity of the majority of spots (Fig. S2B) was similar to that found for mono-dispersed, individual, Cy3B fluorophores viewed under identical conditions on a coverslip in vitro (Fig. S2B). Finally, analysis of individual intensity trajectories over time demonstrated that the majority showed single step photobleaching (Fig. S2B). However, a significant population (40%) showed more complex behavior in which intensity alternated between two intensity levels (see below for further details).


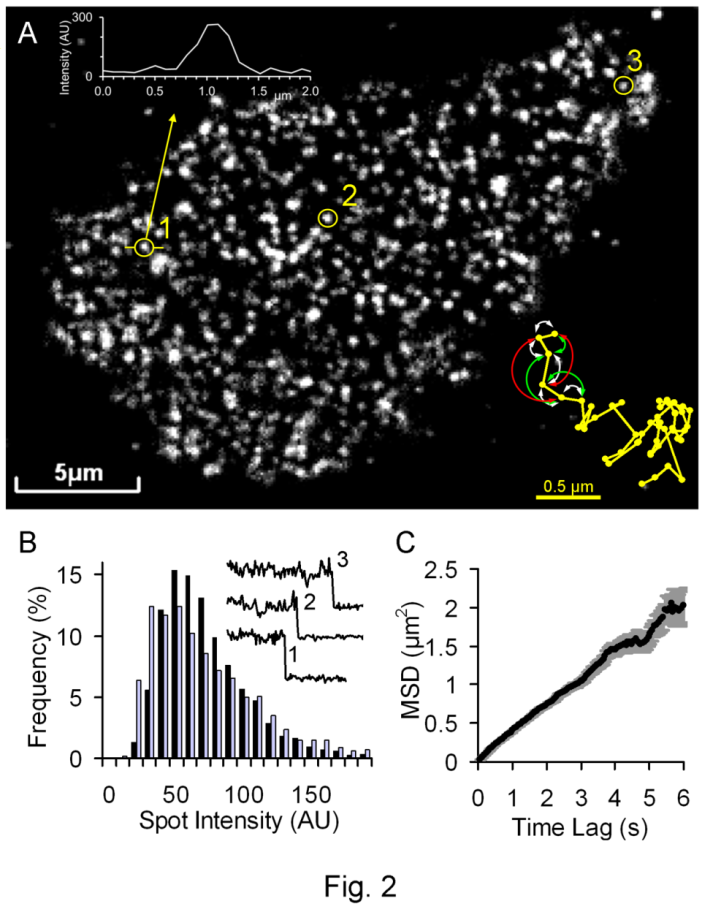


**Figure S2:** Evaluation of the approach to analyze single molecules of M_2_ receptors by TIRFM.

**(A)** A single (30 ms exposure) image of a CHO^M2^ cell labeled with Cy3B-telenzepine imaged under TIRF microscopy. **Top Left Insert** – The intensity profile of an individual Cy3B-telezepine labeled receptor marked by a circle on the main image. **Bottom Right Insert** - the zoomed individual trajectory (yellow) of a single M_2_ molecule. The curved arrows illustrate how the algorithm calculates the MSD as a function of delta time (Δt). For example: the white arrows show pairs of data points, measured at the minimal time interval Δt (30 ms), the green arrows show data pairs at time interval 2Δt (60ms), red arrows at time interval 3Δt (90 ms) and so on. This allows an average MSD vs Δt plot to be created for all possible time values for all objects identified (shown in panel C). **(B)** Distribution of intensities of individual M_2_ containing spots at the beginning of the video records (black columns, 7440 identified objects in 10 cells. The light grey columns show the distribution of the measured intensities of single molecules of Cy3B-telenzepine adhering to a glass coverslip. Because of the very much higher receptor density in HL-1 cells and the consequent overlap of spots (see Fig S4), it was not possible to do the equivalent data analysis at the beginning of a recording from these cells. **Insert** - Three example intensity trajectories of M_2_ molecules marked by yellow circles on panel A. In each case, the intensity of the spot remains at a near constant value and then suddenly falls to background level (typical of about 57% of the trajectories). This behavior, often called “single-step” photobleaching, is characteristic of a single fluorophore molecule. **(C)** Averaged Mean Squared Displacement (MSD ± SEM) versus Δt (time interval) plot for the M_2_ receptor spatial trajectories obtained from video recordings of 13 cells.

***1.8. Discriminating between M_1_ and M_2_ receptor subtype labeling***

Since we know that Cy3B-telenzepine is capable of binding to both M_1_ and M_2_ receptors with high affinity, we confirmed that the major subtype present in cardiac cells is the M_2_ receptor by measuring the extent of inhibition of Cy3B-telenzepine binding following pretreatment with methoctramine (which is an M_2_ selective antagonist; see for example refs [[8](#_ENREF_8), [9](#_ENREF_9)]).

HL-1 cells were incubated with 1μM or 3μM methoctramine for 0.5h, before labeling with 1 nM Cy3B-telenzepine (as above) for 1h. The degree of Cy3B labeling was then quantified at 1 and 18 hours (Fig. S3). The concentrations of methoctramine were chosen, based on reported affinity constants for M_2_ and M_1_ receptors (9), in order to maximise inhibition of M_2_ receptor labeling whilst sparing (relatively) the inhibition at M_1_ receptors. Following methoctramine pretreatment, the proportion of blocked (i.e. methoctramine occupied) M_2_ receptors was predicted to be 88% and 96% versus 42% and 70% for M_1_ receptors (for 1 and 3 µM resp.). We found that the number of Cy3B-telenzepine labeled receptors was 8 ± 4% and 4 ± 2% (for 1 and 3μM treatments respectively, n = 10 cells) compared to untreated cells (see Fig S3 for data at 1h). Similar levels of inhibition were observed at 18h. These results are consistent with the overwhelming majority (>95%) of receptors being of subtype M_2_.

At 18h the level of Cy3B-telenzepine labeling in control and methoctramine pretreated cells had decreased by 50% giving an estimated half-life of Cy3B-telenzepine dissociation of ~18h.


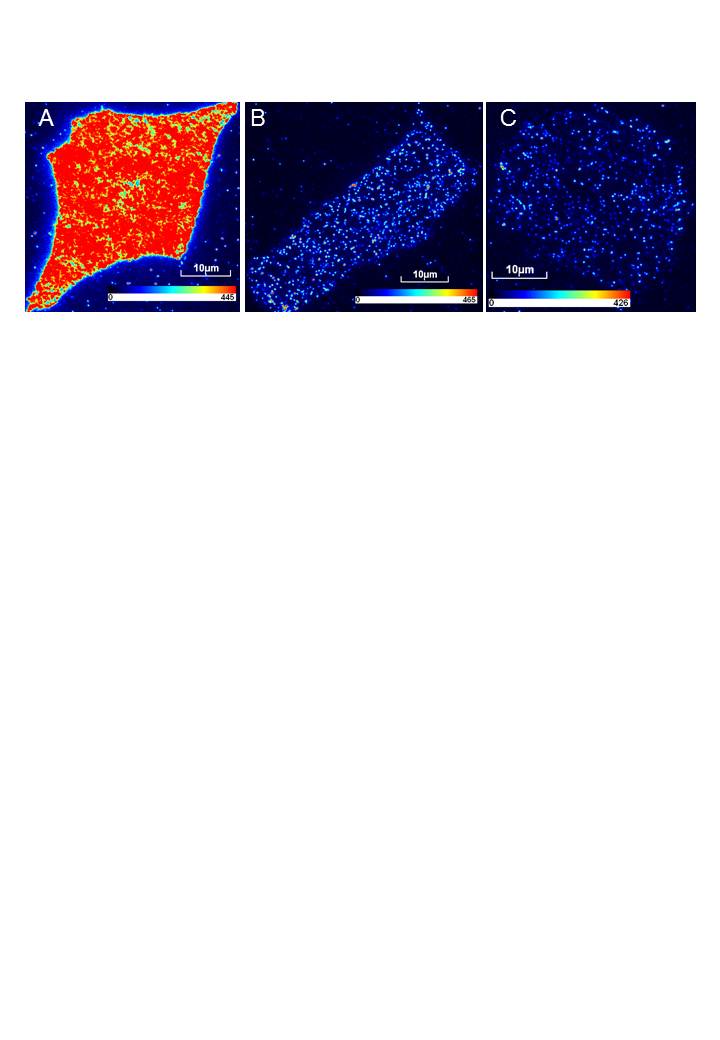


**Figure S3:** Effect of methoctramine pre-treatment on muscarinic receptor labeling in HL-1 cells (Panel A= 0; B= 1μM; C= 3μM). The data shown represent typical cells measured 1h after addition of Cy3B-telenzepine. Methoctramine binds more tightly to M_2_ than M_1_ receptors and the dramatic decrease in receptor labeling observed in panels B and C is consistent with >95% of the receptors being of the M_2_ subtype.

**2. Data analysis and modelling**

***2.1. Image Analysis***

An automated single particle tracking (ASPT) algorithm [[10](#_ENREF_10)] implemented in custom-written software, GMimPro ([www.nimr.mrc.ac.uk/gmimpro/](http://www.nimr.mrc.ac.uk/gmimpro/)), was used to identify and track individual fluorescent spots on sequential video frames. Output from this image analysis gave fluorophore centroid positions (with 25 nm precision), integrated fluorophore intensities and total fluorophore number. Spatial trajectories (i.e. positions vs. time or “tracks”) and intensity trajectories (i.e. intensities vs. time) were then further analyzed to determine: 1) the lateral diffusion coefficient, D_lat_, which was derived from averaged mean squared displacement (MSD) versus time interval (Δt) plots; 2) average fluorophore density and distribution across the cell membrane; 3) intensity distribution of individual spots and intensity fluctuations with time (i.e. observation and counting of stepwise photobleaching events).

Because individual receptors can only be identified as discrete spots of light when their diffraction-limited images do not overlap, we found spot counting by ASPT only gave reliable estimates of density, ρ, when there were <0.8 fluorescent spots per square micron. For randomly dispersed objects, the expected mean distance between nearest neighbor objects (4ρ)^-0.5^ = 780 nm [[11](#_ENREF_11)]. We therefore exploited the fact that fluorophore number, decays monotonically with time due to photobleaching. This enables the initial (or starting) density, ρ_0_, to be calculated by extrapolation of spot counts back to the start of the illumination period.

This approach to spot counting and estimation of receptor density was validated by measuring the bulk photobleaching rate from the change in integrated image intensity (summed over the whole cell) with time, compared to the decay in spot counts at densities below 0.8 µm^-2^ and comparing our experimental results with those from simulated data sets generated using realistic noise levels, and known fluorophore densities. Illumination start time was established by initiating camera recording before the cell was moved into the illumination region. During the experiments, the photobleaching rate was ~0.15 s^‑1^ (i.e. t_½_ ≈ 5 s).

***2.2. Estimation of initial receptor density from spot counting and bulk photobleaching***

At spot densities above 0.8 μm^-2^, average inter-spot distances fall close to the optical diffraction-limit (~300 nm) resulting in overlap of the point spread functions. This means that individual spots are no longer reliably identified. Fig. S4 (lower panels) shows image snap-shots of a region of interest (“ROI”) of an HL-1 cell at various times during a video record. The red boxes indicate putative spots identified by the ASPT algorithm. At early times in the video sequence (t < 10 s) the spot density is so high that individual spots overlap and their images coalesce. However, as the sample photobleaches the spot density falls and the average inter-spot distance gradually increases to the point where individual spots are readily and reliably identified (e.g. for video images at t ≥ 10 s)

Using a Monte-Carlo simulation to generate “fake-data” with exactly the same noise, signal and photobleaching characteristics as our real video data, we found that the spot count value obtained using our ASPT analysis saturates at around 1.5 spots.μm^‑2^ (Fig. S4). During all of our experiments reported in the main paper, zero time (i.e. t=0 on the graphs) was determined by initiating video image capture before moving to the cell region so that the start time of illumination (and therefore the onset of photobleaching) could be determined. The video records could then be synchronized (to the illumination start time, t=0).

The spot density (i.e. number of receptors per square micron) could be determined by calculating the relationship between bulk fluorescence intensity and spot count density by fitting data obtained after photobleaching had caused spot density to fall below the threshold value of 0.8 μm^-2^ and then extrapolating back to t=0. In the example shown (Fig. S4), we show modeled, “fake data” in color using values of 2, 5, 10, 15 fluorophores.μm^-2^, compared to an example “real data” set obtained from an HL-1 cell (in black). The real data lies between modeled densities of 5 and 10 fluorophores.μm^‑2^. The solid lines, which overlay the spot count data, after the density falls below the threshold value of ~0.8 spots.μm^-2^, are the summed intensity of the entire cell, which is plotted on the right-hand axis. We used a least squares fitting procedure to determine the relationship between bulk cell fluorescence and spot density (at spot density<0.8 μm^-2^) so that the starting spot density could be calculated by extrapolation to t=0.


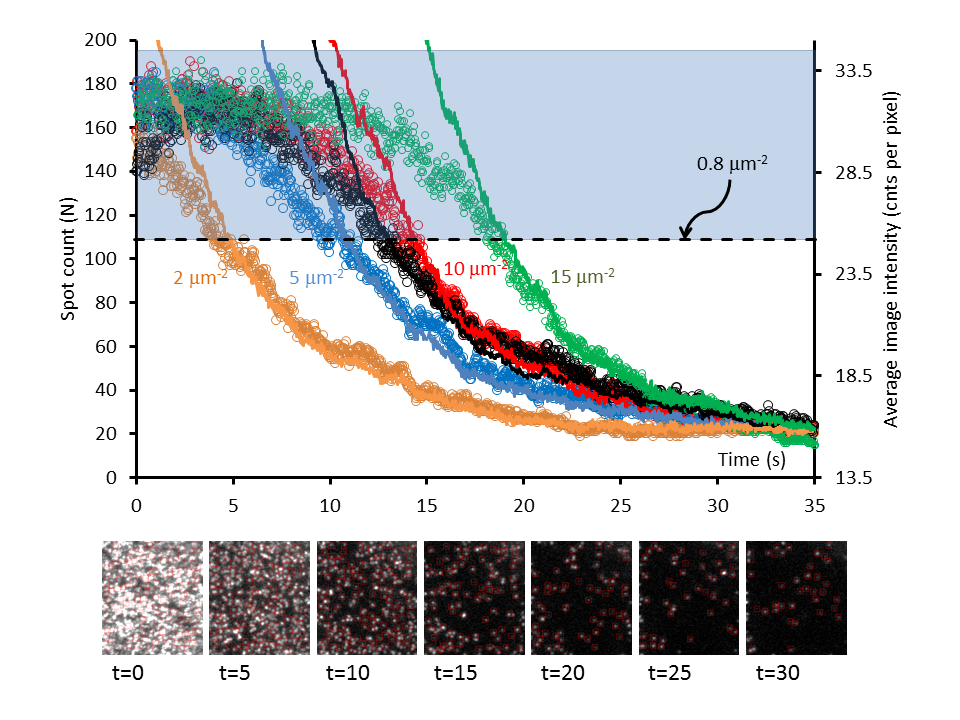


**Figure S4:** Upper graphs show the approach used to estimate spot densities above 0.8 μm^‑2^. Lower montage of images are single video frames of a region of interest (ROI = 12 μm x 12 μm = 144 μm^2^) of an HL-1 cell viewed by TIRF microscopy. The bright spots are Cy3B-telenzepine fluorophores. At the start of the record (t=0) the density of spots is so high that the ASPT algorithm fails to identify all the individual spots (as many spots overlap in position) the apparent spot count value is 165, density = 1.15 μm^‑2^ (see black graph above at t=0). At later times, individual spots are readily identified (e.g. at t=15 s, spot count =100, density = 0.7 μm^‑2^). To validate our ASPT algorithm and investigate the saturated spot count value we simulated “fake-data” using a Monte-Carlo simulation seeded with different starting spot densities (colored lines). Note that the HL-1 cell data lies between the simulations of 5 and 10 spots.μm^-2^. The solid lines are the integrated intensity of the entire ROI – plotted against the right hand scale. We used a least squares fitting algorithm to obtain the relationship between spot intensity counts (where ρ < 0.8 μm^-2^) as a function of integrated fluorescence intensity so that the initial spot could be estimated by extrapolation to t=0 (see text).

***2.3. Analysis of intensity trajectory track types***

Intensity trajectories for individual fluorescent spots were analyzed for stepwise changes in fluorescence intensity by an edge-detection algorithm which used the 1^st^ derivative of the spot intensity vs. time (following a 3-point running average (smoothing) of the intensity data and a 5-point running estimate of the derivative) to identify sudden changes in spot intensity. Data before and after each transition point was averaged to obtain an estimate of the mean intensity during the plateau periods (Fig. S5). Each intensity trajectory was then assigned to a particular “track type” (S6A) and results were histogrammed to see which track types predominate (Fig. S6B). The dominant track types (i.e. histogram peaks) are shown highlighted in red in Fig. S6A.

**Figure S5**: Analysis of step-wise changes in spot intensity trajectories: Each intensity trajectory (in this example red dots show spot intensity vs. time) was fitted to a series of step-wise transitions (e.g. black line) using the first derivative of the data (blue line) to detect sudden changes in intensity (see text for details). The intensity trajectory of every spot detected in the video sequence was analyzed as shown in this example so that each intensity trajectory could then be assigned to a particular “track type”. The example shown here is a track of “Type 2” (see below).


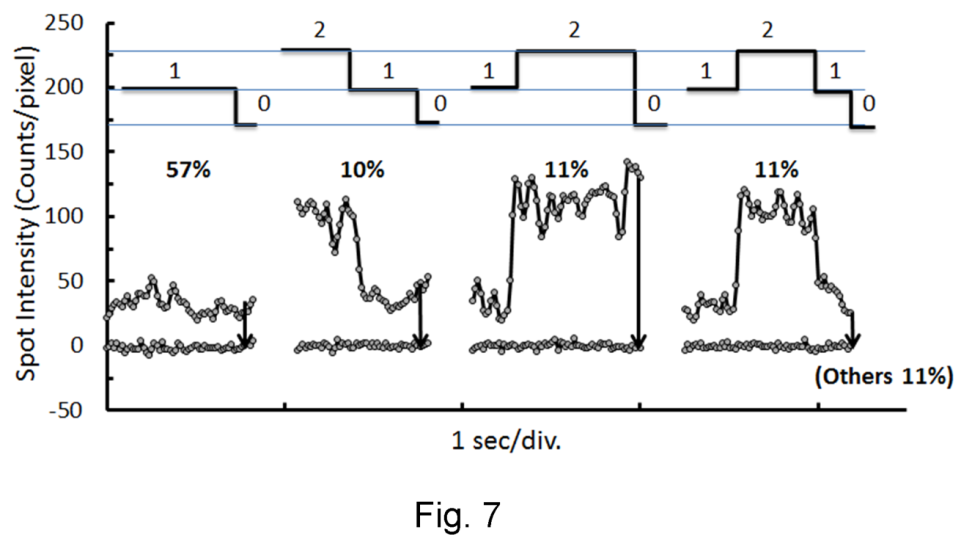


1

2

3

6


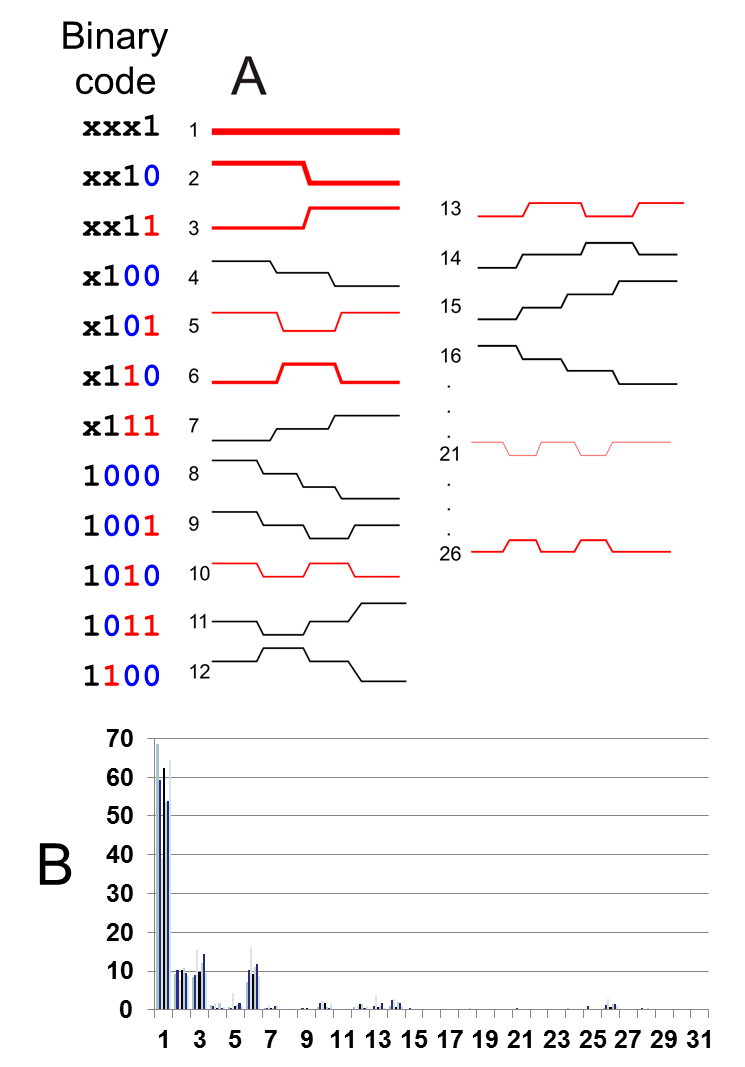


**Figure S6:** The coding system used to characterize each intensity track type.

**(A)** “Type 1” tracks remain at roughly constant intensity then fall in a single step to baseline (compatible with single step photobleaching). NOTE: the final transition to baseline present in every track is not shown; “Type 2” tracks exhibit two stepwise transitions before disappearing; “Type 3” tracks exhibit an initial constant level which rises suddenly to a higher level before falling in a single step to baseline (disappearing). The decimal coding value is obtained by moving forward through time for each intensity trajectory, starting with an initial score of 1 then multiplying the cumulated value by 2 for each negative transition detected or multiplying by 2 and adding 1 for each positive (upwards transition) detected. The track types highlighted in red can be explained by the expected behavior of a single fluorophore (i.e. monomeric receptor) (track type 1) or by two fluorophores (i.e. transient dimer formation and dissociation)

**(B) Left -** Histogram showing the distribution of intensity track types measured for 7 cardiomyocyte cells. **Right** - Example data illustrating the predominant track types (1, 2, 3, 6).

***2.4. Relationship between the bulk expression level of muscarinic receptors (nmoles.g^‑1^ protein) and the surface density of the receptors (molecules.μm^-2^)***

***2.4.1. CHO cells***

The molecular weight of M_2_ receptors (non-glycosylated) is 51.7 kDa, which corresponds to a specific activity of 19.3 μmoles.g^‑1^ receptor protein. The specific activity of the M_2_ receptor preparations in the CHO membranes used is in the range 1-3 nmoles.g^‑1^ total membrane protein. Assuming any internal membrane-bound (i.e. non-cytoplasmic) receptors are present at the same level as at the plasma membrane, it would be expected that the plasma membrane would have a density of (1 to 3) / 19,300 or ~(0.5 to 1.5) / 10,000 of the level of close packed receptors. The effective observed close packed density of rhodopsin from AFM [[12](#_ENREF_12)] is 48,000 ± 8,000 monomers.μm^-2^. [Note: The highest possible packing density is estimated to be 69,000 monomers.μm^-2^]. Assuming that the maximum packing of M_2_ receptors is comparable to that observed for rhodopsin (i.e. they have a similar cross-sectional area, despite having a 34% greater M_R_), it would be expected that the density of the receptors on the membrane would be in the range: 48,000 x (0.5 to 1.5) / 10,000 monomers.μm^-2^, i.e. 2 to 7 μm^-2^.

Given the multiple assumptions in this calculation, our observed density of objects that we measure (~3 ± 1 μm^-2^, Table S1, cardiomyocytes) is in reasonable agreement with this estimate. It should be noted that receptor expression in individual CHO cells is not the same for all cells, despite it being a clonal cell line. There is considerable variation, both in size and expression levels.

***2.4.2. Mouse heart***

The observed levels of muscarinic receptors in the mouse heart are ~0.05 to 0.1 nmole.g^‑1^ membrane protein [[13](#_ENREF_13), [14](#_ENREF_14)]. Therefore, using the same argument as in the previous section, one expects 0.1 to 0.2 objects.μm^‑2^ assuming that all cardiac cells express muscarinic receptors at the same level. We in fact observe very heterogeneous expression levels, with the majority of cells not expressing detectable levels of muscarinic receptors. Those cells expressing muscarinic receptors do so at a level of ~1.8 ± 0.4 μm^-2^ (Table 1)

Our data are therefore compatible with a small percentage (5 to 10%) of mouse cardiac myocytes expressing muscarinic receptors. This has not been previously recognized.

***2.5. Monte Carlo simulations of receptor-receptor collisions***

Knowing receptor density and rate of lateral diffusion we performed a Monte Carlo simulation to estimate the collision rate of receptors at the membrane. Receptors were randomly seeded on a 2-dimensional surface at different starting densities (see Fig. S7) and then each molecule performed an independent Brownian walk using a step size that was well below the molecular dimension (mean step size was ~0.1nm per time interval). Collisions were detected and scored at each time step when any pair of molecular coordinates came within 6nm of each other. The model was allowed to run for sufficient time to give reliable estimates of collision rate. The simulation was coded using IgorPro Ver6.0.


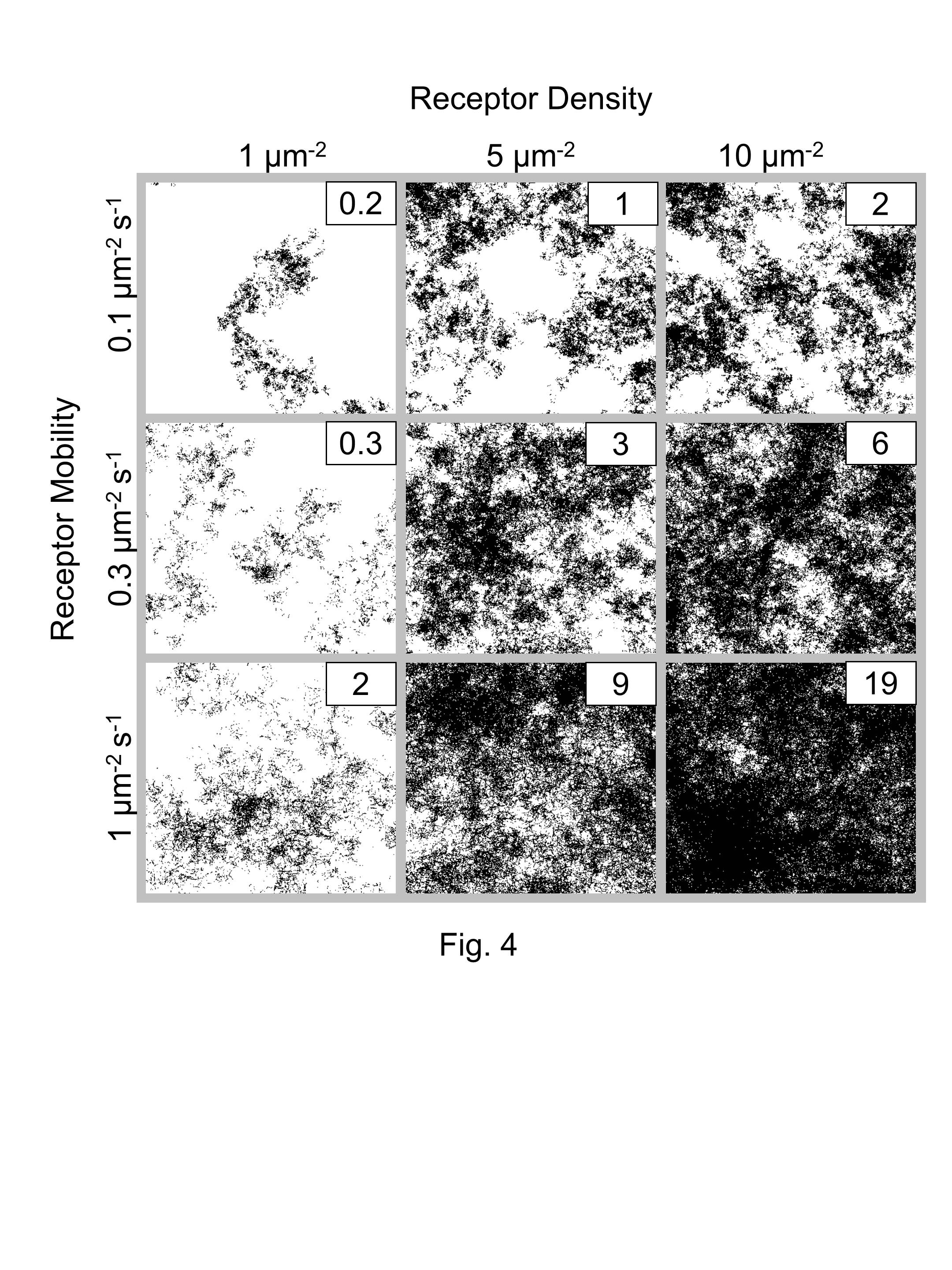


**Figure S7:** A Monte-Carlo computer simulation of receptor movement showing how the frequency of collisions depends on mobility and density of the receptors. Each panel shows the modeled trajectories of receptors within a 1×1 µm^2^ area; the model time step was 0.1 μs, and total run time 1 s. At low receptor densities the individual tracks are clearly visible, but at higher receptor density or higher receptor mobility the tracks overlap significantly; a consequence of the higher collision rate. The average number of collisions (per receptor per second) are shown in the boxes at the corner of each panel. Model values, close to those measured in the present study, are shown in the central box.

**References**

[1] Buckley NJ, Bonner TI, Buckley CM, Brann MR. Antagonist binding properties of 5 cloned muscarinic receptors expressed in CHO-K1 cells. Mol Pharmacol. 1989;35:469-76.

[2] Claycomb WC, Lanson NA, Stallworth BS, Egeland DB, Delcarpio JB, Bahinski A, et al. HL-1 cells: A cardiac muscle cell line that contracts and retains phenotypic characteristics of the adult cardiomyocyte. Proc Natl Acad Sci U S A. 1998;95:2979-84.

[3] Lazareno S, Dolezal V, Popham A, Birdsall NJM. Thiochrome enhances acetylcholine affinity at muscarinic M_4_ receptors: Receptor subtype selectivity via cooperativity rather than affinity. Mol Pharmacol. 2004;65:257-66.

[4] Nobles M, Sebastian S, Tinker A. HL-1 cells express an inwardly rectifying K^+^ current activated via muscarinic receptors comparable to that in mouse atrial myocytes. Pflugers Arch. 2010;460:99-108.

[5] Lazareno S, Gharagozloo P, Kuonen D, Popham A, Birdsall NJM. Subtype-selective positive cooperative interactions between brucine analogues and acetylcholine at muscarinic receptors: Radioligand binding studies. Mol Pharmacol. 1998;53:573-89.

[6] Mashanov GI, Tacon D, Knight AE, Peckham M, Molloy JE. Visualizing single molecules inside living cells using total internal reflection fluorescence microscopy. Methods. 2003;29:142-52.

[7] Hern JA, Baig AH, Mashanov GI, Birdsall B, Corrie JET, Lazareno S, et al. Formation and dissociation of M_1_ muscarinic receptor dimers seen by total internal reflection fluorescence imaging of single molecules. Proc Natl Acad Sci U S A. 2010;107:2693-8.

[8] Lazareno S, Birdsall NJM. Pharmacological characterization of acetylcholine-stimulated [35S]-GTPγS binding mediated by human muscarinic m1-m4 receptors: antagonist studies. Br J Pharmacol. 1993;109:1120-7.

[9] Birdsall NJM. Muscarinic acetylcholine receptors. The IUPHAR Compendium of Receptor Characterization and Classification. 2nd Edition ed: UPHAR Media, London 2000. p. 54-63.

[10] Mashanov GI, Molloy JE. Automatic detection of single fluorophores in live cells. Biophys J. 2007;92:2199-211.

[11] Clark PJ, Evans FC. Distance to nearest neighbour as a measure of spatial relationships in populations. Ecology. 1954;35:445-53.

[12] Fotiadis D, Liang Y, Filipek S, Saperstein DA, Engel A, Palczewski K. Atomic-force microscopy: Rhodopsin dimers in native disc membranes. Nature. 2003;421:127-8.

[13] Ito Y, Kato A, Fujino T, Okura T, Yoshida K, Nanri M, et al. Muscarinic receptor binding and plasma drug concentration after the oral administration of propiverine in mice. Lower Urinary Tract Symptoms. 2010;2:43-9.

[14] Gomeza J, Shannon H, Kostenis E, Felder C, Zhang L, Brodkin J, et al. Pronounced pharmacologic deficits in M_2_ muscarinic acetylcholine receptor knockout mice. Proc Natl Acad Sci U S A. 1999;96:1692-7.
